# Psychological capital mediates the relationship between medication adherence and cancer-related fatigue in breast cancer patients undergoing long-term treatment

**DOI:** 10.3389/fpsyt.2025.1615271

**Published:** 2025-07-01

**Authors:** Chunli Yan, Yane Chu

**Affiliations:** ^1^ Department of Oncology, The Third Xiangya Hospital of Central South University, Changsha, Hunan, China; ^2^ Department of Nursing, The Third Xiangya Hospital of Central South University, Changsha, Hunan, China; ^3^ School of Basic Medicine, Changsha Medical University, Changsha, Hunan, China

**Keywords:** breast cancer, psychological capital, medication adherence, cancer-related fatigue, mediating effect, long-term pharmacotherapy, positive psychology, mental health

## Abstract

**Background:**

Breast cancer is one of the most prevalent malignant tumors among women worldwide. Although long-term pharmacological treatment has substantially improved survival rates, it is often accompanied by psychological burdens, including cancer-related fatigue (CRF) and diminished adherence to therapy. CRF is a pervasive and debilitating symptom that adversely affects physical functioning and emotional well-being. Psychological capital (PsyCap), a construct encompassing self-efficacy, hope, optimism, and resilience, has been shown to enhance treatment engagement and promote mental health. However, its role as a potential psychological mediator between medication adherence and CRF is yet to be thoroughly investigated.

**Objective:**

To investigate the mediating role of PsyCap in the relationship between medication adherence and CRF in patients with breast cancer undergoing long-term treatment.

**Methods:**

A total of 100 breast cancer patients admitted between June 2022 and June 2024 were recruited using convenience sampling. Data from 90 valid responses were analyzed. Participants completed the PsyCap Questionnaire (PCQ-24), Self-Reported Medication Adherence Rating Scale (SR-MARS), and CRF Scale (CFS). Pearson’s correlation analysis was used to assess associations among variables. A mediation analysis was conducted using the bootstrap method with 5,000 resamples.

**Results:**

The mean scores for PsyCap, medication adherence, and CRF were 86.65 ± 8.37, 5.36 ± 1.12, and 36.77 ± 5.98, respectively. PsyCap was positively correlated with medication adherence (*r* = 0.994, p < 0.05) and negatively correlated with CRF (*r* = –0.992, p < 0.05). Medication adherence was also negatively correlated with CRF (*r* = –0.994, p < 0.05). Mediation analysis confirmed that PsyCap significantly mediated the relationship between medication adherence and CRF (indirect effect = 0.357, 95% CI did not include zero), accounting for 55.68% of the total effect.

**Conclusion:**

PsyCap partially mediated the association between medication adherence and CRF. Interventions aimed at enhancing PsyCap may improve adherence and reduce CRF in breast cancer patients receiving long-term pharmacotherapy.

## 1 Introduction

Breast cancer, one of the most frequently diagnosed malignancies among women, continues to pose a major global public health challenge. According to recent global statistics, more than 2 million new cases and approximately 680,000 breast cancer-related deaths occur annually, with projections reaching 3 million new cases and 1 million deaths by 2040 (1). Although advances in early diagnosis and treatment have led to improved survival rates, the prolonged nature of breast cancer therapy imposes considerable psychological and emotional burdens on patients. Emerging evidence indicates that, beyond the physical impact of the disease, breast cancer survivors frequently experience substantial mental health challenges, including anxiety, depression, and psychological distress throughout the course of treatment (2, 3). Cancer-related fatigue (CRF), a persistent, subjective sense of tiredness associated with cancer or its treatment, is one of the most debilitating psychological and somatic symptoms, negatively affecting patients’ cognitive function, mood, social interactions, and quality of life (4–6).

Pharmacotherapy is a cornerstone of breast cancer management and often spans years, especially for hormone receptor–positive subtypes. Sustained adherence to long-term endocrine or targeted therapies is essential for achieving favorable outcomes, including recurrence prevention and survival benefits (7). However, long-term medication use often presents adherence challenges owing to side effects, psychological distress, and treatment fatigue (8, 9). Psychological resilience and mental resources play a pivotal role in shaping patients’ medication behaviors and symptom trajectories.

Psychological capital (PsyCap), a construct rooted in positive psychology, comprises four core psychological components: self-efficacy, optimism, hope, and resilience. These internal resources have been associated with enhanced psychological adjustment, greater engagement in health-promoting behaviors, and improved emotional regulation among individuals with chronic illnesses (10). Although prior research has explored the association between PsyCap and specific cancer outcomes, little is known about its mediating role in the relationship between medication adherence and CRF among patients with breast cancer undergoing long-term pharmacotherapy.

Therefore, this study aimed to investigate the mediating effect of PsyCap on the relationship between medication adherence and CRF in breast cancer patients. By identifying this psychological pathway, we seek to offer new insights into mental health–informed interventions that could support treatment adherence and reduce psychological distress during extended pharmacological treatment.

## 2 Materials and methods

### 2.1 Participants

A total of 100 female patients diagnosed with breast cancer between June 2022 and June 2024 were recruited using convenience sampling. All participants and their family members provided written informed consent prior to enrollment. The study protocol was approved by the hospital’s Medical Ethics Committee.

The inclusion criteria were as follows: (1) fulfillment of the diagnostic criteria outlined in the Guidelines and Specifications for Breast Cancer Diagnosis and Treatment issued by the Chinese Anti-Cancer Association (2021) (11); (2) pathologically confirmed diagnosis of breast cancer; (3) age ≥18 years; (4) high treatment compliance and ability to cooperate with the study procedures; and (5) no current infectious diseases.

Exclusion criteria were: (1) presence of other malignant tumors, (2) diagnosis of brain disease, (3) documented cognitive impairment, (4) presence of uncontrolled or chronic pain, and (5) advanced breast cancer (stage IV) with distant metastasis.

### 2.2 Instruments and measures

#### 2.2.1 General information questionnaire

A self-designed demographic and clinical questionnaire was used to collect general information from participants. The items included age, marital status, educational attainment, disease duration, pathological stage, and whether the patient had received chemotherapy or radiotherapy or not. Prior to administration, all participants and their families were provided with a standardized explanation of the study’s purpose, procedures, and instructions to ensure comprehension and voluntary participation. The researchers were trained to clarify any patient questions without influencing their responses. A total of 100 questionnaires were distributed, of which 93 were returned. After excluding 3 invalid responses, 90 valid questionnaires were included in the final analysis, yielding a response rate of 90.0%.

#### 2.2.2 Psychological capital

The Chinese version of the Psychological Capital Questionnaire-24 (PCQ-24), originally developed by Luthans et al. (12), has demonstrated excellent reliability and cultural suitability for the long-term assessment of PsyCap in Chinese populations. The scale measures four dimensions of positive psychological resources–self-efficacy, optimism, hope, and resilience–with six items per subscale. Each item is rated on a 6-point Likert scale ranging from 1 (strongly disagree) to 6 (strongly agree), resulting in a total score ranging from 24 to 144. Higher scores reflect higher levels of PsyCap. The scale demonstrated excellent internal consistency in this study, with a Cronbach’s alpha coefficient of 0.947.

#### 2.2.3 Medication adherence

A Self-reported medication Adherence Rating Scale (SR-MARS) was used to assess patient medication adherence. This questionnaire includes 10 items: medication-taking frequency, reason for missing doses, trust in medical instructions, punctuality of medication timing, accuracy of dosage, comfort with medication use, confidence in medication, psychological state during medication use, self-adjustment of medication, and medication storage management. Each item scores 0–1 with a total score of 0-10. Details are shown in the **Supplementary File**. Higher scores indicate better medication adherence. Cronbach’s alpha coefficient for the SR-MARS in this study was 0.75, indicating good reliability.

#### 2.2.4 Cancer-related fatigue

CRF was measured using the cancer-related fatigue Scale (CFS), originally developed by Okuyama et al. (13). The scale consists of 15 items covering three dimensions: physical fatigue, emotional fatigue, and cognitive fatigue. Items are rated on a 5-point Likert scale ranging from 1 (not at all) to 5 (very much), with item 5 reverse-scored. The total scores range from 15 to 75, with higher scores indicating more severe fatigue. CFS has been used in clinical and research settings in China and showed high internal consistency in this study (Cronbach’s α = 0.88), suggesting its potential reliability and cultural adaptability for assessing cancer-related fatigue in the Chinese populations.

### 2.3 Data processing and statistical methods

All statistical analyses were performed using SPSS version 26.0 (IBM Corp., Armonk, NY, USA). Descriptive statistics were used to summarize the demographic and clinical characteristics, and categorical variables are presented as frequencies and percentages. Pearson correlation coefficients were calculated to assess the correlations between PsyCap, medication adherence, and CRF. Mediation analyses were conducted using PROCESS macro version 3.4, and structural equation modeling (SEM) was performed using AMOS version 25.0 to evaluate the hypothesized mediating pathways. The significance of the indirect effects was tested using the bias-corrected bootstrap method with 5,000 resamples. A two-tailed p-value < 0.05 was considered statistically significant for all analyses.

## 3 Results

### 3.1 Participant characteristics

Ninety patients breast cancer completed the study. The mean age of the participants was 52.36 years (SD = 7.62), ranging from 42 to 65 years. The average disease duration was 15.29 months (SD = 3.58), with a range of 5–22 months.

Most participants were married (n = 82, 91.11%), while 8 (8.89%) were unmarried. In terms of education, 36 participants (40.00%) had completed high school or had attained a higher level of education, whereas 54 (60.00%) had an education level of junior high school or below.

Postoperative treatment distribution revealed that 67 patients (74.44%) received chemotherapy and 23 patients (25.56%) underwent radiotherapy. Regarding pathological staging, 37 participants (41.11%) were classified as stage I, 48 (53.33%) as stage II, and five (5.56%) as stage III.

### 3.2 Scores of PsyCap, medication adherence, and CRF

As shown in **Table 1**, the mean PsyCap score among the 90 patients with breast cancer patients was 86.65 (SD = 8.37). The average score for medication adherence was 5.36 (SD = 1.12), while the mean score for CRF was 36.77 (SD = 5.98).

**Table 1:** Mean scores for psychological capital, medication adherence, and cancer-related fatigue among breast cancer patients (n = 90).

Variable	Number of items	Mean ± SD (score)
Psychological capital (PCQ-24)	24	86.65 ± 8.37
– Self-efficacy	6	19.67 ± 4.25
– Optimism	6	21.77 ± 5.67
– Hope	6	20.53 ± 4.68
– Resilience	6	20.86 ± 4.39
Medication adherence (SR-MARS)	10	5.36 ± 1.12
Cancer-related fatigue (CFS)	15	36.77 ± 5.98

Higher scores indicate greater levels of psychological capital and medication adherence, but more severe cancer-related fatigue.

### 3.3 Correlation analysis among PsyCap, medication adherence, and CRF

**Figure 1:**
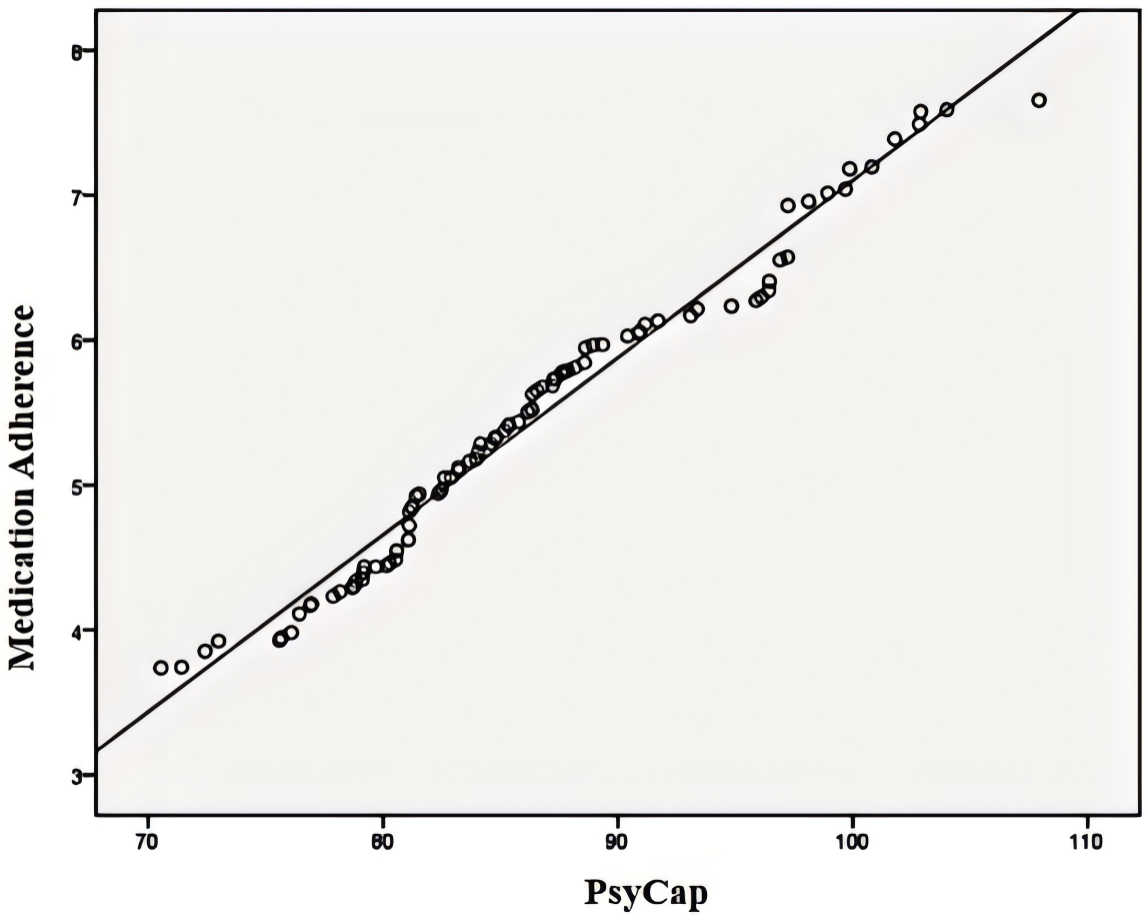
Correlation between psychological capital and medication adherence. CRF, cancer-related fatigue.

**Figure 2:**
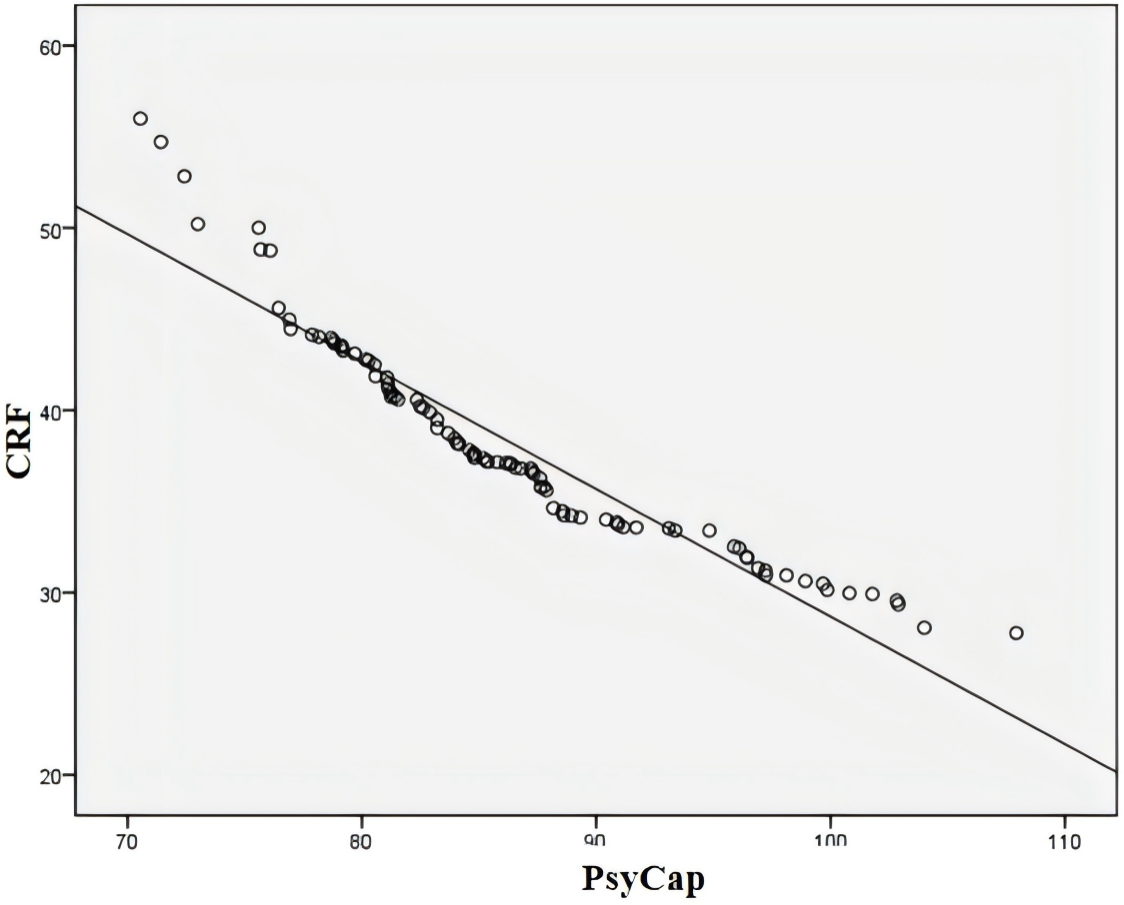
Correlation between psychological capital and cancer-related fatigue. CRF, cancer-related fatigue.

**Figure 3:**
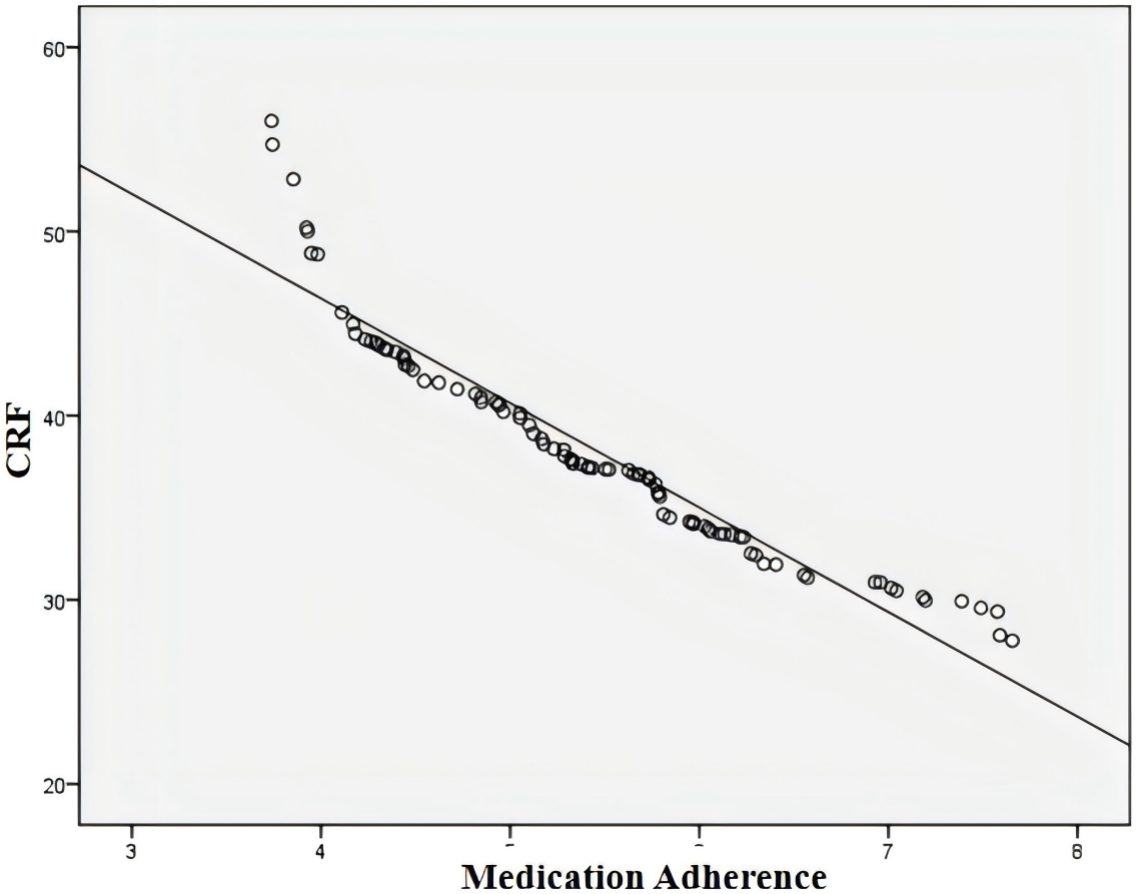
Correlation between medication adherence and cancer-related fatigue. CRF, cancer-related fatigue.

As shown in **Table 2**, PsyCap was positively correlated with medication adherence (*r* = 0.994, p < 0.05), indicating that higher levels of PsyCap were associated with better adherence to pharmacotherapy. Additionally, PsyCap was negatively correlated with CRF (*r* = –0.992, p < 0.05), suggesting that individuals with higher PsyCap experienced less fatigue. Similarly, a significant negative correlation was observed between medication adherence and CRF (*r* = –0.994, p < 0.05), indicating that patients with better adherence reported lower levels of fatigue (**Figures 1**–**3**).

**Table 2:** Correlation matrix among psychological capital, medication adherence, and cancer-related fatigue.

Scale	Total psychological capital	Self-efficacy	Optimism	Hope	Resilience	Medication adherence	Cancer-related fatigue
Total psychological capital	1.000	–	–	–	–	–	–
Self-efficacy	0.991	1.000	–	–	–	–	–
Optimism	0.982	0.977	1.000	–	–	–	–
Hope	0.988	0.992	0.982	1.000	–	–	–
Resilience	0.989	0.983	0.994	0.986	1.000	–	–
Medication Adherence	0.994	0.997	0.982	0.994	0.988	1.000	–
Cancer-related fatigue	-0.992	-0.990	-0.987	-0.988	-0.987	-0.994	1.000

All correlations are significant at p < 0.05.

### 3.4 Mediating effect of PsyCap between medication adherence and CRF

**Figure 4:**
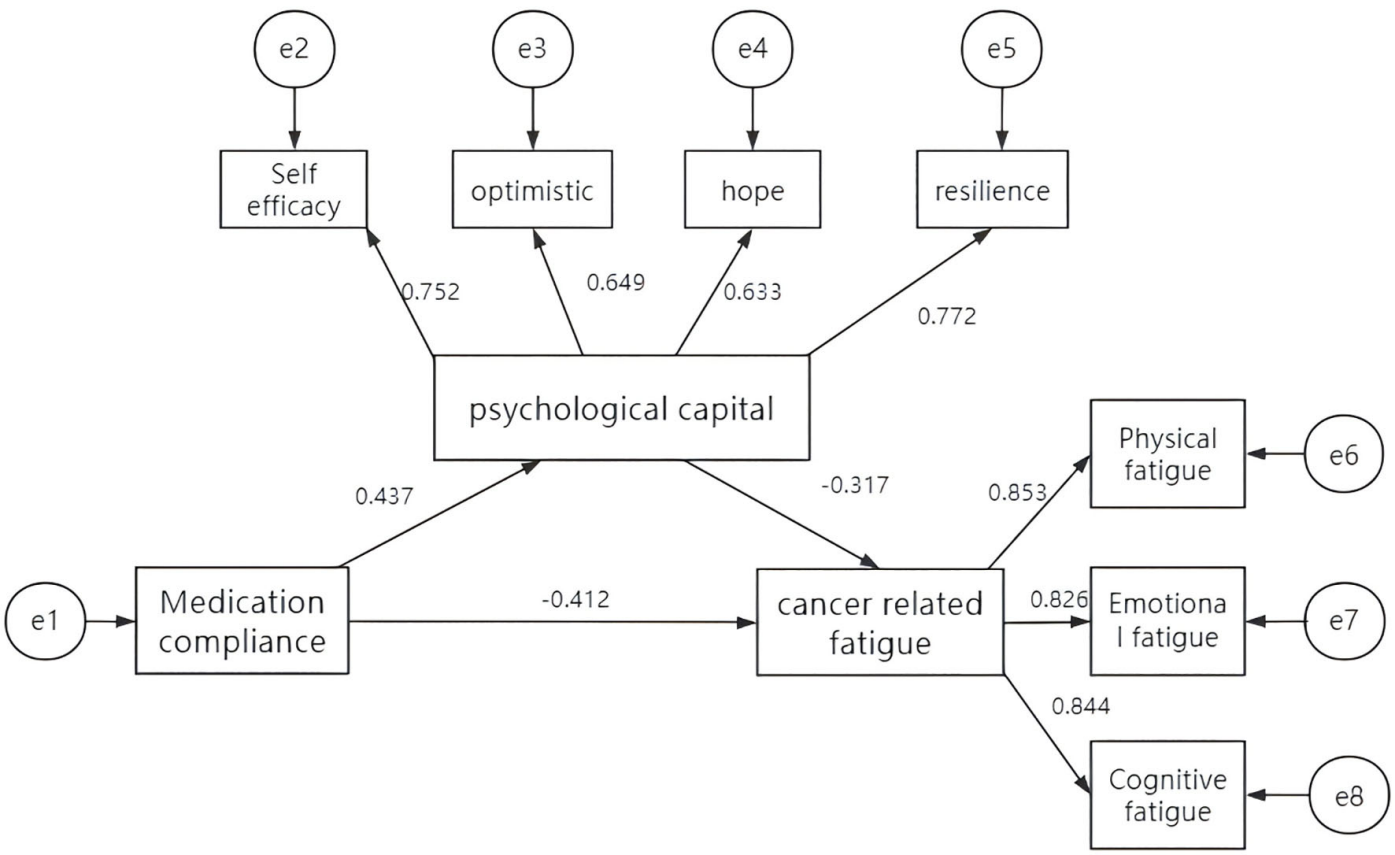
The pathway of the mediating effect of psychological capital between medication adherence and cancer-related fatigue.

A structural equation model (SEM) was constructed to examine the mediating role of PsyCap in the relationship between medication adherence and CRF among patients with breast cancer. In this model, medication adherence was specified as the independent variable, CRF as the dependent variable, and PsyCap as the mediating variable. Model estimation and testing were conducted using AMOS version 25.0 (**Figure 4**).

The model demonstrated a good overall fit: χ²/df = 1.425, root mean square error of approximation (RMSEA) = 0.047, goodness-of-fit index (GFI) = 0.974, adjusted goodness-of-fit index (AGFI) = 0.926, comparative fit index (CFI) = 0.973, normed fit index (NFI) = 0.953, and Tucker–Lewis’s index (TLI) = 0.947. All the standardized path coefficients were statistically significant.

To further evaluate the mediation effect, bootstrapping with 5,000 resamples was applied using a bias-corrected confidence interval (CI) approach. The 95% CI for the indirect effect did not contain zero, confirming the statistical significance of the mediating role of PsyCap in the pathway from medication adherence to CRF. Detailed results are presented in **Tables 3**, **4**.

**Table 3:** Path coefficients for the mediating role of psychological capital between medication adherence and cancer-related fatigue.

Path	Coefficient	95% CI	p-value
PsyCap ← Medication Adherence	0.437	0.274 – 0.635	< 0.001
CRF ← PsyCap	-0.317	-0.355 – -0.427	< 0.001
CRF ← Medication Adherence	-0.412	-0.518 – -0.563	< 0.001

PsyCap, psychological capital; CRF, cancer-related fatigue.

**Table 4:** Direct, indirect, and total effects of psychological capital as a mediator.

Effect type	Effect value	Standard error	95% CI	p-value	Proportion of total effect (%)
Indirect Effect	0.357	0.039	0.135 – -0.479	< 0.001	55.68
Direct Effect	0.326	0.028	0.308 – -0.757	< 0.001	44.32
Total Effect	0.563	0.059	0.459 – -0.898	< 0.001	–

## 4 Discussion

Breast cancer is a highly heterogeneous malignant tumor with a complex pathogenesis and is often influenced by genetic factors (14). Previous studies have shown that approximately 5% of breast cancer cases are associated with BRCA1, BRCA2, and other high-penetrance gene mutations (15, 16). In recent years, treatment strategies have transitioned from single-modality approaches to multidisciplinary comprehensive care (17, 18). Beyond disease management, increasing attention has been directed toward the psychological burden experienced by patients. Studies have shown that fatigue, sleep disturbance, and psychological symptom clusters, comprising anxiety, depression, tension, irritability, sadness, and worry, are among the most common symptom clusters experienced by patients with breast cancer. Moreover, the composition and severity of these symptom clusters often vary across the different stages of cancer treatment (19). CRF is a distressing and persistent symptom prevalent across the continuum of diagnosis, treatment, and recovery, with reported incidence rates ranging from 70% to 100% (20).

Despite the availability of effective therapies, medication adherence among breast cancer patients remains suboptimal. Studies have estimated that approximately 28% of patients exhibit some level of nonadherence (21). Irregular medication use can result in sub-therapeutic drug concentrations, limiting efficacy, increasing the risk of tumor recurrence, and compromising long-term outcomes.

Psychological capital, first proposed by Luthans et al., is rooted in positive psychology and reflects an individual’s positive mental state, encompassing self-efficacy, optimism, hope, and resilience (22). While prior research has explored the relationships between PsyCap and individual health outcomes, such as fatigue, few studies have examined the mediating role of medication adherence and CRF in oncology populations. The present study addresses this gap by offering evidence that PsyCap significantly mediates the relationship between adherence and fatigue, thus providing a novel framework for intervention development.

Each PsyCap component contributes uniquely to treatment engagement and psychological resilience. Self-efficacy refers to the belief in one’s ability to execute behaviors necessary to manage illness, whereas optimism reflects a positive attributional style. Hope promotes goal-directed motivation and resilience denotes the capacity to recover from adversity (23). In this study, the mean PsyCap score among participants was 86.65 ± 8.37, reflecting a moderate-to-low level consistent with the findings of Han et al. (24). The relatively low PsyCap levels observed may stem from the emotional impact of cancer diagnosis, including fear of disease progression and physical decline, which can deplete psychological resources.

Medication adherence was also found to be moderate (5.36 ± 1.12) and likely influenced by fluctuating psychological states. Patients often show high adherence early in treatment, which is driven by hope for recovery. However, as treatment progresses, particularly in the absence of visible improvements, negative emotions, such as anxiety and despair, may reduce motivation and disrupt medication routines. Additionally, fatigue and prolonged treatment can erode medication management.

Effective pharmacological therapy suppresses tumor growth and controls disease progression. However, poor adherence disrupts this process, which leads to disease progression and increased fatigue. The mean CRF score in this study was 36.77 ± 5.98, indicating a moderate level of severity, consistent with the findings of Lapidari et al. (25). Biologically, fatigue may be driven by tumor-derived metabolites, such as cytokines and lactic acid, which disrupt neurotransmitter balance and trigger systemic inflammation. Psychologically, cancer is a major stressor. Negative coping mechanisms such as avoidance and denial may intensify emotional exhaustion and energy depletion.

Correlation analysis revealed that PsyCap was positively associated with medication adherence and negatively associated with CRF. Similarly, adherence was inversely correlated with CRF. The mediation analysis confirmed that PsyCap partially mediated the relationship between medication adherence and CRF, accounting for 55.68% of the total effect. This suggests that patients with higher PsyCap are more confident in their treatment, adhere more consistently to medication regimens, and are better equipped to manage their fatigue.

Psychological interventions that enhance PsyCap, such as psychoeducation, cognitive-behavioral strategies, and positive reinforcement, could significantly improve adherence and symptom management. The Saudi Arabian Primary Care Asthma Assessment and Management Program emphasizes the importance of early screening for psychological symptoms and treatment adherence in patients with poorly controlled asthma to prevent further deterioration of symptoms (26). Fuentes et al. (27) reported that psychological stressors negatively influenced medication adherence among Latino individuals living with HIV. Similarly, Chayadi et al. (28) demonstrated that anxiety and depression in cancer patients can exacerbate CRF. Mindfulness-based interventions for patients with cancer have been shown to alleviate psychological distress and promote physical well-being. Additionally, a study conducted in the United States (29) identified emotional symptoms, sleep disturbances, and neuromuscular fatigue as significant predictors of CRF, highlighting their relevance in developing targeted medical support strategies for patients experiencing cancer-related fatigue.

Gu et al. (30) demonstrated that increasing PsyCap improves treatment confidence and adherence. Similarly, Xin et al. (31) reported that web-based positive psychotherapy can elevate PsyCap and alleviate CRF. Interventions that foster self-efficacy, encourage hope, and cultivate resilience may help patients reframe fatigue as a temporary and manageable challenge, promoting proactive coping strategies, such as seeking support, engaging in physical activity, and practicing relaxation techniques.

These findings demonstrate a potential positive feedback cycle: elevated psychological capital contributes to improved medication adherence, which, in turn, alleviates symptom burden and further enhances psychological well-being. This highlights the need for integrative psychosocial care in oncology practice rather than focusing exclusively on pharmacological treatment or physical symptoms. It is essential for healthcare teams to systematically assess and support patients’ psychological resources throughout the course of long-term cancer treatment.

This study has several limitations. First, all participants were recruited from a single tertiary care hospital in China, which may limit the generalizability of the findings to a broader or more diverse population. Second, the relatively small sample size (n = 90) reduced the statistical power and may have introduced sampling bias. Third, its cross-sectional design precludes causal inferences. Future research should adopt a longitudinal or interventional design to confirm the mediating role of PsyCap over time. Expanding the study to include multicenter samples and diverse cultural backgrounds would enhance external validity. Third, the SR-MARS was a self-developed medication adherence scale. Although it has been applied in our clinical practice, further validation studies are needed to establish its reliability and validity.

In addition to medication adherence and CRF, the PsyCap of patients with breast cancer is influenced by multiple physiological and lifestyle factors, including dietary habits and overall physical condition. Diets high in sugar and fat have been shown to disrupt gut microbiota diversity, thereby reducing the synthesis of brain-derived neurotrophic factor (BDNF), impairing hippocampal neurogenesis, and diminishing patients’ sense of hope and cognitive control. Conversely, restrictive dieting or nutrient deficiencies, particularly inadequate intake of vitamin D and omega-3 fatty acids, can contribute to mitochondrial dysfunction, potentially leading to chronic fatigue syndrome and initiating a negative cycle of “malnutrition → fatigue → psychological exhaustion.”

Although chemotherapy can effectively delay disease progression in breast cancer, it also produces adverse effects that may undermine psychological resilience. For instance, chemotherapy-induced alopecia not only alters appearance but may also provoke catastrophic thinking related to disease progression. Additionally, estrogen depletion resulting from endocrine therapy may dysregulate serotonin transporter function, thereby triggering depressive symptoms and exacerbating psychological distress.

Clinically, integrated interventions addressing the interconnection among diet, physical health, and psychological well-being may enhance patients’ PsyCap. A multidimensional and interdisciplinary intervention framework is warranted, which combines physiological, psychological, and social support components and involves collaboration among patients, families, healthcare professionals, and broader environmental systems. Targeted cognitive behavioral therapy and mindfulness-based stress reduction techniques can help patients manage distress by fostering present-moment awareness and reducing avoidance behaviors. Nutritional plans should be tailored to individual symptoms and needs, while physical rehabilitation interventions such as manual lymphatic drainage, compression garment use, and limb function training can be employed to address physical impairments. Family involvement in treatment discussions can foster shared decision making and enhance patients perceived control. Importantly, coordinated interventions led by interdisciplinary teams, including psychologists, nutritionists, and rehabilitation therapists, are essential for ensuring the professionalism and effectiveness of PsyCap-enhancing strategies.

## 5 Conclusion

This study demonstrated that PsyCap partially mediates the relationship between medication adherence and CRF in patients with breast cancer. Higher PsyCap is associated with better adherence and reduced fatigue, suggesting that interventions aimed at enhancing PsyCap may serve as effective strategies for improving treatment outcomes. It is recommended that the PsyCap scale be implemented in oncology specialty clinics to facilitate baseline screening of PsyCap among newly diagnosed patients and long-term medication users. Patients identified as high risk, defined by a PsyCap score below 24, should be prioritized for intervention, and PsyCap enhancement strategies should be integrated into standard diagnostic and treatment pathways. Given the mental health challenges inherent in long-term cancer pharmacotherapy, integrating psychological support into standard oncology care may foster resilience, adherence, and overall wellbeing. Future multicenter longitudinal studies are warranted to validate and expand upon these findings.

## Data Availability

The original contributions presented in the study are included in the article/**Supplementary Material**. Further inquiries can be directed to the corresponding author.
