# Supplementary material for: Psychological capital mediates the relationship between medication adherence and cancer-related fatigue in breast cancer patients undergoing long-term treatment
Source: Front Psychiatry. 2025 Jul 1;16:1615271. doi: 10.3389/fpsyt.2025.1615271 (PMC12259705; doi:10.3389/fpsyt.2025.1615271)
Supplement: Supplementary file 1 [file Table1.docx]

Supplementary File

**Self-Reported Medication Adherence Scale (SR-MARS)**

Please answer the following questions based on your medication use over the past [time period, e.g., one month]. Choose the option that best describes your situation (single choice per question).

**Medication-taking frequency**

□ Never missed a dose

□ Occasionally missed (≤2 times/week)

□ Frequently missed (3–5 times/week)

□ Missed almost every day

**Reason for missing doses**

□ Forgot

□ Felt symptoms had improved

□ Worried about side effects

□ Ran out of medication and did not refill

□ Other: _____________

**Trust in medical instructions**

□ Fully trust and strictly follow

□ Mostly trust but occasionally question

□ Frequently doubt the effectiveness or necessity

**Punctuality of medication timing**

□ Always on time (within ±30 minutes)

□ Mostly on time (within ±2 hours)

□ Often off schedule (more than ±2 hours)

□ Take it at random times

**Accuracy of dosage**

□ Never took the wrong dose

□ Occasionally took the wrong dose (≤2 times/month)

□ Frequently took the wrong dose (≥1 time/week)

**Comfort with medication use**

□ No adverse reactions

□ Occasional adverse reactions

□ Frequent adverse reactions

**Confidence in medication**

□ Strongly believe it helps relieve symptoms

□ Somewhat lack confidence in its effectiveness

□ No confidence in its effectiveness

**Psychological state during medication use**

□ Good

□ Average

□ Poor

**Self-adjustment of medication**

□ Never stop or reduce dosage on my own

□ Occasionally adjust (e.g., when symptoms improve)

□ Frequently adjust on my own

**Medication storage management**

□ Store according to medical advice/instructions

□ Store randomly
